# Microscale communication between bacterial pathogens and the host epithelium

**DOI:** 10.1038/s41435-021-00149-1

**Published:** 2021-09-29

**Authors:** Ann-Kathrin Mix, Griseldis Goob, Erik Sontowski, Christof R. Hauck

**Affiliations:** 1 Lehrstuhl Zellbiologie, Fachbereich Biologie, Universität Konstanz, Konstanz, Germany; 2 Konstanz Research School Chemical Biology, Universität Konstanz, Konstanz, Germany

**Keywords:** Bacterial infection, Infection

## Abstract

Pathogenic bacteria have evolved a variety of highly selective adhesins allowing these microbes to engage specific surface determinants of their eukaryotic host cells. Receptor clustering induced by the multivalent microorganisms will not only anchor the bacteria to the tissue, but will inevitably trigger host cell signaling. It has become clear, that these bacteria-initiated signaling events can be seen as a form of localized communication with host epithelial cells. Such a microscale communication can have immediate consequences in the form of changes in host cell membrane morphology or cytoskeletal organization, but can also lead to transcriptional responses and medium- and long-term alterations in cellular physiology. In this review, we will discuss several examples of this form of microscale communication between bacterial pathogens and mammalian host cells and try to delineate their downstream ramifications in the infection process. Furthermore, we will highlight recent findings that specialized pathogenic bacteria utilize the adhesin-based interaction to diffuse the short-range messenger molecule nitric oxide into the host tissue. While anti-adhesive strategies to disrupt the initial bacterial attachment have not yet translated into medical applications, the ability to interfere with the microscale communication emanating on the host side provides an unconventional approach for preventing infectious diseases.

## Introduction

Microorganisms can tightly interact with host tissues by employing dedicated proteins, so-called adhesins, to engage surface structures of their target cells. In many cases, these specific binding events determine not only the cell type tropism, but also the species specificity of the infecting organism. A structural and biochemical understanding of these molecular interactions is helpful to elucidate the details of such specific binding events. However, to appreciate the role of these interactions during the infection process it is important to unravel the functional consequences downstream of the adhesin–receptor interplay. Indeed, the engagement of host surface structures by bacterial adhesins goes well beyond the mere physical association. The multivalent nature of the adhesin-covered bacterial surface will result in receptor clustering and will initiate intracellular signaling in eukaryotic cells. In that way, bacteria can orchestrate, from the outside, a multitude of intracellular responses ranging from cytoskeletal rearrangements, changes in membrane morphology, and vesicle trafficking to modulation of gene expression. Seen from this perspective, it becomes obvious that the choice of host receptor(s) targeted by bacterial adhesins can be decisive for all downstream processes in the interaction between pathogens and their host. An emerging theme in this field is the recognition that the tight interaction afforded by adhesin-mediated contact also sets the stage for the exchange of additional bacterial products, often transferred by dedicated translocation systems or provided in the form of membrane permeable intermediates of bacterial metabolism such as short chain fatty acids (SCFAs) or even gaseous messengers such as nitric oxide (NO). In this review, we will discuss several examples, where the initial adhesin-mediated contact is the pre-requisite for further exchange and molecular communication on a microscale. We hope that these representative cases will illustrate the sophisticated interplay occurring at the microbe-epithelial interface and guide the way to innovative measures to intercept bacterial infections at this early point.

## Host responses elicited by adhesin–receptor binding

Surface expressed adhesins are mostly unique for a given bacterial species and encompass integral membrane proteins (e.g., classical beta-barrel outer membrane proteins (OMPs) such as neisserial Opa proteins or *Haemophilus* OMP P1 [1, 2]) as well as complex extracellular macromolecular assemblies (e.g. such as fimbriae and pili [3, 4]). These differences in the molecular make up also form the basis for the classic separation in fimbrial adhesins, which are associated with surface appendages visible in electron microscopy and variously termed pili, fimbriae, or fibrils, versus the so-called afimbrial adhesins. For fimbria and pili, the adhesin proper is often only a minor component of the heteromeric, multisubunit protein complexes, which make up the hair-like extracellular structure. A classic example for these types of adhesive structures are the P-pili of uropathogenic *Escherischia coli* (UPEC), which are mainly build by the structural protein PapA [5]. Localized at the tip of P-pili are the minor pilus subunits PapE, PapF, PapK, and PapG, with the actual adhesin, PapG, at the far distal tip of the fibril. PapG has lectin-like properties and binds to galactose-α(1–4)-galactose-containing glycolipids [6]. With few exceptions, most fimbrial adhesins operate as lectins and recognize specific glycan moieties found on various glycoproteins and glycolipids of their host organisms. For example, more than 35 distinct fibrillar adhesins have been identified so far in the genomes of pathogenic *E. coli* strains isolated from different host organisms [7]. Testing a selection of these adhesins on glycan arrays demonstrated that their binding specificity for particular carbohydrate linkages matches the occurrence of these structures in their respective hosts [8]. However, as these glycan structures usually are contained within different glycoproteins and glycolipids alike, binding to glycan residues usually does not trigger a uniform intracellular signaling response, but rather engages a variety of different surface determinants on the infected cell. Exceptions are seen in situations, where few, highly abundant glycoproteins are present on particular host cell types. One well-described example in this regard is the fimbrial tip-associated adhesin FimH encoded by many UPEC strains [9]. FimH binds to α-D-mannosylated glycoproteins, which are highly abundant on the bladder epithelium in the form of uroplakins [10]. Uroplakin Ia (UPIa) together with additional uroplakins is embedded into lipid rafts of the urothelial plasma membrane [11]. FimH engages UPIa in a catch-bond manner triggering a conformational change of the uroplakin complex [12–14] resulting in the casein kinase II-mediated phosphorylation of threonine residues in the UPIIIa cytoplasmic tail [15]. These modifications of the bacteria-engaged receptor translate into stimulation of defined intracellular signaling cascades such as activation of phosphatidylinositol 3-kinase (PI3K) accompanied by local phosphatidylinositol 3,4,5-triphosphate (PIP_3_) and calcium elevation [16], phosphorylation of FAK, and complex formation between α-actinin and vinculin [17]. Together, these receptor-triggered intracellular signals are responsible for the observed actin cytoskeleton rearrangements, which result in plasma membrane zippering around UPEC and UPEC bladder invasion [16].

In contrast to the mostly glycan-binding fimbrial adhesins, such a highly focused activation of intracellular signaling as summarized here for FimH-triggered uroplakin signaling is usually a hallmark of afimbrial adhesins. Indeed, afimbrial adhesins often engage a single, specified host receptor via direct protein-protein interactions. In that sense, afimbrial adhesins on the bacterial surface are ideally suited to cluster a specified receptor and trigger a defined signaling pathway (see next chapter and Table 1). Though fimbrial adhesins are less common in gram-positive microorganisms, several pathogens including *Streptococcus pyogenes*, *Streptococcus* *agalactiae*, *Streptococcus* *pneumoniae*, *Corynebacterium diphtheriae*, or *Staphylococcus aureus* also produce fibrillar surface appendages. The fimbrial proteins are secreted via the Sec pathway and processed by sortase enzymes, which covalently link the pilin subunits and attach the fimbrial base to peptidoglycan [18, 19]. Similar to pili of gram-negative bacteria, also pili of gram-positive bacteria consist of major structural proteins and dedicated adhesins. For example, the pilus of *S. pneumoniae* harbors the minor pilus subunit RrgA, which functions as the adhesin and which has homologues in other pathogenic streptococci such as *S. pyogenes* or *S. agalactiae* [20–22]. Interestingly, elucidation of the RrgA crystal structure revealed that the adhesin harbors an integrin I domain-like binding site for extracellular matrix proteins such as collagen [23]. Furthermore, RrgA forms a polybasic, cradle-shaped surface, which might allow recognition of acidic carbohydrate structures present in the form of sulfated glycosaminoglycans in host target tissues [23]. Therefore, the 94 kDa RrgA protein combines carbohydrate recognition (as found in fimbrial adhesins of gram-negative bacteria) with a specific protein-protein interaction module (as found in afimbrial adhesins of gram-negative bacteria).

**Table 1:** Selected bacterial adhesins and their target structures in human tissues.

Adhesin class	Pathogen	Adhesin (adhesive structure)	Ligand/Receptor	Anti-adhesive strategy	References
Fimbrial adhesin	*Corynebacterium diphtheriae*	SpaA (SpaA-type pilus)	Collagen		[81]
	*Escherichia coli* (UPEC)	FimH (type I pilus)	α-D-mannosylated glycoproteins, CEACAM6	Mannoside M4284	[9, 10, 12–14, 77]
	*Escherichia coli* (UPEC)	PapG (Pap pilus)	Galactose-α(1-4)-galactose-containing glycolipids	Galabioside	[5, 6, 82]
	*Streptococcus agalactiae*	Gbs1478 (PilA); Gbs104	Collagen, fibronectin		[20]
	*Streptococcus pneumoniae*	RrgA (Rrg pili)	Fibronectin, collagen, laminin		[22, 23]
	*Streptococcus pyogenes*	Cpa	Collagen		[21]
Afimbrial adhesin	*Acinetobacter baumannii*	Ata (trimeric autotransporter)	Collagen, laminin	Small molecule inhibitor	[30, 83]
	*Bartonella henselae*	BadA (trimeric autotransporter)	Collagen, fibronectin, laminin	Small molecule inhibitor	[29, 83]
	*Escherichia coli* (UPEC)	Afa/Dr	CEACAM1,5		[56]
	*Fusobacterium nuceatum*	FadA	E-Cadherin, VE-Cadherin		[48, 49]
	*Haemophilus influenzae*	Omp P1	CEACAM1,3,5		[2]
	*Helicobacter pylori*	HopQ	CEACAM1,3,5,6		[55]
	*Leptospira interrogans*	CbpF (trimeric autotransporter)	VE-Cadherin	Small molecule inhibitor	[46, 47, 83]
	*Listeria monocytogenes*	InlA	E-Cadherin		[40, 41]
	*Moraxella catarrhalis*	UspA1 (trimeric autotransporter)	CEACAM1,3,5	Small molecule inhibitor	[28, 53, 54, 83, 84]
	*Neisseria gonorrhoeae*	Opa_CEA_	CEACAM1,3,5,6		[1, 85, 86]
	*Neisseria meningitidis*	Opa_CEA_	CEACAM1,3,5,6		[1, 86]
	*Neisseria meningitidis*	NadA (trimeric autotransporter)	LOX-1	Small molecule inhibitor	[83, 87]
	*Streptococcus agalactiae*	β protein	CEACAM1,5		[57]
	*Streptococcus pneumoniae*	PsaA	E-Cadherin	Small molecule inhibitors	[45, 88, 89]
	*Yersinia enterocolitica*	Invasin	Integrin β1		[36, 90, 91]
	*Yersinia enterocolitica*	YadA (trimeric autotransporter)	Collagen, fibronectin, laminin		[26, 31, 32]
	*Yersinia pseudotuberculosis*	Invasin	Integrin β1		[36, 90, 91]
	*Yersinia pseudotuberculosis*	YadA (trimeric autotransporter)	Collagen, fibronectin, laminin		[31, 32]

## Microbe-induced host cell responses by afimbrial adhesins

Besides pilus- or fimbriae-associated adhesins, numerous pathogens employ afimbrial adhesins to bind to host cells and stimulate signaling processes. While afimbrial adhesins can be tightly integrated into the bacterial outer membrane, often they consist of elongated molecules, which place the host-cell-binding site at a distance from the bacterial surface. Examples for this type of afimbrial adhesins are the trimeric autotransporter adhesins (TAAs) also termed oligomeric coiled-coil adhesins. Here, the spacing of the globular, amino-terminal head domain away from the bacterial surface is accomplished by a stalk-like coiled-coil domain [24]. Members of this family of adhesins are found in numerous gram-negative bacteria [25]. A common feature of these adhesins resides in their carboxy-terminus, which is embedded into the outer membrane and functions as an autotransporter to shuttle the passenger part (stalk and head domain) to the bacterial surface [25]. Prominent and well-studied TAAs are YadA of *Yersinia enterocolitica* [26], NadA of *Neisseria meningitidis* [27], UspA1 of *Moraxella catarrhalis* [28], BadA of *Bartonella henselae* [29], or Ata of *Acinetobacter baumannii* [30]. The host targets of the *Yersinia* adhesin A (YadA) have been studied in detail, as this adhesin is tightly connected to *Yersinia* virulence. Similar to other TAAs, the head domain of YadA engages in a direct protein–protein interaction with extracellular matrix proteins including collagen, fibronectin, and laminin [31, 32]. Thereby, the bacteria can indirectly engage host cell integrins and trigger integrin signaling. Indeed, infection of different cell types with *Yersinia* or YadA-expressing *E. coli* activates known integrin-triggered cytoplasmic signaling modules including focal adhesion kinase and phosphatidylinositol(PI)-3′ kinase [33–35]. In addition to YadA, *Y. pseudotuberculosis* and *Y. enterocolitica* express a second afimbrial surface adhesin, which targets integrins and which has been termed invasin, as it triggers the actin cytoskeleton-dependent internalization of *Yersinia* into non-phagocytic cells. In contrast to YadA, invasin directly engages the integrin β1 subunit with 100-fold higher affinity than the physiological integrin ligands fibronectin, collagen, or laminin [36]. Despite this extraordinary affinity, invasin triggers similar integrin-initiated intracellular signaling events such as activation of focal adhesion kinase and PI-3′ kinase [37, 38].

Both, invasin- and YadA-mediated binding will position the microorganism close to the host cell membrane, allowing type III secretion system-mediated translocation of effector molecules [39]. Such translocated effectors are optimized to alter cellular functions from within the infected host cell. In this context, it becomes apparent that the adhesin-mediated contact helps to precipitate the bacterial effector secretion onto a single host cell. This aspect of intimate host cell association afforded by afimbrial adhesins will also be discussed further in the next section.

When we now turn to additional examples of afimbrial bacterial adhesins, it becomes obvious that similar to integrins, other mammalian cell adhesion proteins, such as cadherins and immunoglobulin-related cell adhesion molecules (IgCAMs), are also preferred targets for bacterial pathogens. The extraordinary prevalence of cell adhesion proteins to serve as adhesin receptors surely relies on their prominent surface exposure on the host cell. Moreover, mammalian adhesion proteins are often conserved, essential components of the host, where evasion from pathogen binding, e.g., by mutation of adhesin binding sites, would not be compatible with their physiological role. Most importantly, cell adhesion proteins in general are functionally connected to the intracellular actin cytoskeleton affording bound bacteria with a means to orchestrate cytoskeletal organization. This is nicely exemplified in the case of *Listeria monocytogenes*, a gram-positive bacterium, which causes the foodborne disease listeriosis. In the first step of infection, the afimbrial, cell wall attached adhesin InternalinA (InlA) of *L. monocytogenes* binds to E-cadherin [40]. InlA associates with the EC1 domain of human, but not murine or rat E-cadherin and induces caveolin-dependent E-cadherin clustering [41, 42]. Receptor clustering triggers Src-dependent phosphorylation of tyrosine residues within the E-cadherin cytosolic tail, which results in the recruitment of Hakai, a E3 ubiquitin-ligase [43]. Hakai then decorates E-cadherin with an ubiquitin mark, initiating clathrin-dependent endocytosis and actin remodeling (via the Arp2/3 complex) to promote bacterial internalization by a zipper-like mechanism [44]. As discussed above, the cytoplasmic linkage of the cell-cell adhesion molecule E-Cadherin is here again used to the advantage of the bacterium, as it provides a direct conduit to locally modulate actin dynamics and to trigger endocytosis.

Besides *L. monocytogenes*, also other bacteria have been reported to interact with human cadherins such as *S. pneumoniae* [45]*, Leptospira interrogans* [46, 47], or *Fusobacterium nucleatum* [48, 49]. By associating with E-cadherin, *F. nucleatum* also activates the E-cadherin–β‐catenin signaling pathway leading to elevated nuclear translocation of β‐catenin and activation of lymphoid enhancer factor/T-cell factor-mediated transcription. In that way, *F. nucleatum* promotes expression of c-myc and cyclin D1, drivers of the eukaryotic cell cycle, leading to enhanced in vitro and in vivo growth of E-cadherin-expressing human colorectal carcinoma cells [49]. Here, the adhesin-initiated microscale communication between a pathogen and the pathogen-associated host cell goes beyond immediate cellular effects, such as membrane organization or cytoskeletal dynamics, but influences cellular gene expression programs. In this regard, the bacteria-induced signaling processes can have a lasting impact on the infected cell and can promote the growth of colorectal tumors. This particular adhesin–receptor interaction also provides one of the few examples, where bacteria positively affect growth of infected host cells, and suggests a mechanistic framework for the observed enrichment of *F. nucleatum* in colorectal carcinoma tissue [50–52].

It is probably not surprising, that engagement of E-cadherin, which is involved in a well-characterized cellular signal transduction cascade, can provide a direct route to modulate gene expression. However, modulation of gene expression in response to adhesin-mediated contact has also been reported for another group of cell adhesion molecules involved in cell–cell adhesion. These receptors belong to the group of IgCAMs. In particular, the carcinoembryonic antigen (CEA)‐related cell adhesion molecules (CEACAMs) provide a docking site for diverse afimbrial adhesins found in a number of human-specific pathogens. CEACAM-binding adhesins have been identified in the pathogenic neisserial species *Neisseria* *gonorrhoeae* and *N. meningitidis* (colony opacity-associated (Opa) proteins) [1], *Moraxella catarrhalis* (the UspA1 protein) [53, 54]*, Haemophilus influenzae* (the Omp P1 protein) [2], *Helicobacter pylori* (the HopQ protein) [55], uropathogenic *E. coli* (the Afa/Dr adhesins) [56], or *S. agalactiae* (the β protein) [57]. As all CEACAM-binding pathogens colonize the mucosal surface and several CEACAM family members can be present on the apical surface of human epithelial cells, it is safe to conclude that the various adhesin-mediated interactions with epithelial CEACAMs have evolved to facilitate mucosal colonization [58]. CEACAMs are known to modulate a number of cellular processes such as cell proliferation, apoptosis, or innate immune responses suggesting a range of opportunities for CEACAM-binding bacteria to influence host responses. Indeed, binding of pathogens to CEACAM1, a family member expressed by some epithelial, endothelial and immune cell types can lead to suppression of innate immune responses providing a plausible explanation for the prevalence of CEACAM-binding human pathogens. However, other CEACAM family members, which do not possess immunomodulatory properties and which dominate epithelial tissues in the intestine and the genital tract, are also targeted by microorganisms. Moreover, these CEACAM family members, including CEA (the product of the *CEACAM5* gene) and CEACAM6, are GPI-anchored proteins, which lack the propensity for direct signal transduction into the host cytoplasm. Nevertheless, several studies have provided strong evidence that also GPI-anchored CEACAMs can serve as a basis to influence host responses and gene expression events, thereby facilitating colonization of the mucosal surface by specialized pathogens. In this context, a surprising twist to the usual communication between bacterium and host has been unveiled recently, which will be detailed in the next section.

## NO as a short-range, cross-kingdom messenger molecule

**Fig. 1:**
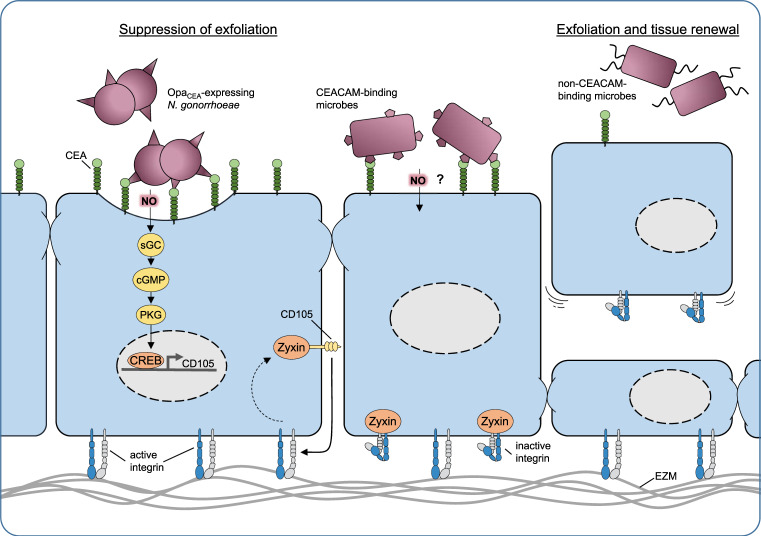
Schematic overview of host cell processes initiated by microbe-derived NO. Host-adapted bacteria (such as Opa_CEA_-expressing *N. gonorrhoeae*) can intimately bind to the apical surface of mucosal epithelial cells, e.g., by engaging members of the human CEACAM family. Under low oxygen conditions, CEACAM-associated gonococci produce nitric oxide (NO) due to nitrite respiration in an anaerobic environment as found in the genital tract. The gaseous radical NO penetrates membranes and can reach the neighboring host cell cytoplasm within a micrometer distance. There, nanomolar concentrations of NO initiate a conserved eukaryotic signaling pathway involving soluble guanylate cyclase (sGC), protein kinase G, and the transcription factor CREB to upregulate CD105 expression. *De novo* expression of CD105, by extracting the adapter molecule zyxin from focal adhesions, leads to increased integrin activity, thereby enforcing the attachment of the infected cell to the extracellular matrix. As a consequence, exfoliation is suppressed (left side) facilitating the colonization of the mucosa. Other CEACAM-binding microbes, such as certain uropathogenic *E. coli*, might also be able to trigger this process (middle). In contrast, microbes, which are not able to trigger this process, stimulate epithelial exfoliation and tissue renewal (right side).

CEACAM-based contact of microbes with epithelial cells triggers enhanced transcription of several genes in the human cells, as revealed more than a decade ago by unbiased microarray-based gene expression analysis [59]. Interestingly, the *de novo* expression of one particular host protein, the Transforming Growth Factor β1-receptor protein CD105 (also known as endoglin), is responsible for a change in the host cell phenotype: expression of CD105 results in altered integrin activity, which in turn leads to enhanced extracellular matrix binding by the infected human epithelial cells [59] (Fig. 1). This increased stickiness of the infected cells to the underlying extracellular matrix translates into a suppression of an efficient innate defense mechanism of the epithelium: the exfoliation. Exfoliation counteracts microbial infection by shedding the bacteria-associated, superficial epithelial cells. This process occurs in stratified epithelia, such as those found in the urogenital tract, but also in single-layered epithelia such as the small intestine, where epithelial cells are constantly shed from the tips of intestinal villi. In turn, the ability to suppress exfoliation facilitates the prolonged colonization of an epithelial surface by bacteria. Indeed, in a humanized mouse model of vaginal infection by *N. gonorrhoeae*, CEACAM-binding gonococcal variants are able to suppress exfoliation in a CEA- and CD105-dependent manner [60, 61]. Obviously, the suppression of exfoliation and the reduced shedding of superficial epithelial cells affords a major advantage to CEACAM-binding microbes during the initial colonization of the mucosa and this mechanism might explain the widespread occurrence of diverse CEACAM-binding adhesins [62]. While knock-down of CD105 demonstrates that this membrane protein holds a central role in this process, it has been unclear, how exactly the bacterial engagement of a GPI-linked CEACAM, such as CEA, can initiate the *de novo* expression of CD105 in the epithelium. The mechanistic details of bacteria-triggered CD105 expression have remained enigmatic, as antibody-mediated clustering of CEA or physiological cell–cell contact based on CEA, in the absence of CEA-binding bacteria, does not lead to CD105 expression [63]. The solution to this conundrum came as a surprise, when it was shown that NO is the key to CD105 expression and the downstream phenotypic change of the infected epithelial cells [63]. Indeed, the membrane permeable gas NO has important conserved physiological signaling functions in eukaryotic cells. Inside mammalian cells, NO reacts with the heme moiety of soluble guanylate cyclases (sGC) leading to increased production of cyclic guanosine-3′-5′-monophosphate (cGMP), which in turn stimulates cGMP-activated protein kinase (PKG). While PKG has multiple cellular targetgs, this enzyme also phosphorylates transcription factors of the cAMP response element-binding protein (CREB) family, which are responsible for enhanced CD105 expression. Pharmacological inhibition of either sGC or PKG or compromising the CREB binding sites in the CD105 promoter prohibit bacteria-triggered increases in CD105 expression and also restore epithelial exfoliation in the presence of CEACAM-binding bacteria [63].

NO is a major intra- and intercellular signaling molecule in mammals, where it is generated by different enzymes of the NO synthase (NOS) family (endothelial NOS (eNOS), inducible NOS (iNOS), or neuronal NOS (nNOS)). In a strictly Ca^2+^ regulated manner, eNOS and nNOS produce nanomolar amounts of NO to control blood pressure and neuronal signaling on a timescale of seconds and minutes. These low concentrations of NO are sufficient to form nitrosyl adducts in proteins that contain a heme moiety such as guanylate cyclase, which is thereby stimulated to form cGMP from GTP [64]. In contrast, iNOS is typically expressed only upon inflammatory stimuli and generates micromolar concentrations of the NO radical over the course of several hours until the enzyme is degraded [65]. Accordingly, the action of iNOS can serve to defend against microbes, as the high NO fluxes produced by iNOS form the basis for the generation of reactive nitrogen oxide species such as dinitrogen trioxide (N_2_O_3_) and peroxynitrite (ONOO^-^), which result in nitrosative and oxidative stress [66]. The strong and constitutive activity of iNOS comes at a cost, as the nitrosative and oxidative stress will also damage the host tissue itself [66].

Interestingly, experiments with NOS inhibitors demonstrated that the NO responsible for increased CD105 expression by infected cells does not originate from endogenous sources [63]. Rather it became apparent, that the NO responsible for the cellular effects in the epithelium originate from bacterial metabolism. In particular, a bacterial nitrite reductase, AniA, is expressed by the pathogens under oxygen-limited conditions and generates NO from nitrite. As a result, a remarkable microscale communication can take place in an anaerobic environment, where low concentrations of the bacterial metabolic product NO are able to trigger physiological signals in mammalian host cells. The diffusible and reactive nature of the NO radical is of particular relevance here: it can readily penetrate neighboring host cells, but due to its reactivity, it is short-lived and has only a limited radius of action [67]. This ephemeral nature of NO can explain the critical role played by the adhesin–receptor interaction in this process. Adhesin-mediated intimate attachment of the bacteria to CEACAMs, while not directly leading to a receptor-triggered signaling cascade, places the bacteria in close contact with the host cell plasma membrane to allow sufficient amounts of NO to reach the cytoplasm of the neighboring epithelial cell. Due to this anchoring function, even a GPI-anchored receptor such as CEA, lacking a cytoplasmic domain, can constitute an essential component in this microbe-host cell communication. The narrow working range of NO also assures that cell-associated bacteria only affect one individual or a small group of cells in the vicinity of the bound pathogen, rather than influencing a larger area of the mucosa. In this respect, NO differs markedly from the mode of action of more stable bacterial metabolites (such as the SCFAs acetate, butyrate, or propionate), which due to their hydrophobic properties and chemical stability can diffuse beyond the epithelial layer [68, 69]. Therefore, microbe-derived SCFAs can affect more distant tissues, which are not in direct contact with bacteria [70, 71]. This implies that stable bacterial metabolites, which reach host tissues, do not reward their producers with an exclusive benefit, but rather allow other microbes to profit from bystander effects. In contrast, the spatial restriction afforded by the short-lived NO radical appears to be highly advantageous in this context, as competing organisms of other pathogenic or commensal bacterial species cannot hijack the benefits of NO production, even if they are co-inhabiting the same ecological niche. Especially in the context of exfoliation, the release of NO and its focused effect on the associated host cell optimizes the colonization fitness of individual or small groups of bacteria, while other microbes are not able to profit from the consequences of NO release. The action of NO seems to be confined to such an extent that even pili, which bind over a distance of several micrometers, might space the bacteria at too far a distance to enable productive NO diffusion [63].

Using NO as a short-range messenger is a remarkable solution for communicating with the host also for a further reason: in contrast to protein-based bacterial factors that trigger host responses, NO as an intermediate product of the denitrification pathway comes with a very low metabolic cost. Bacterial proteins or other macromolecules as secreted effectors require significantly more resources for production and delivery. Indeed, in the case of NO, nanomolar concentrations are sufficient to initiate canonical sGC-PKG signaling in mammalian cells as observed in diverse physiologic processes such as blood pressure regulation or platelet aggregation [64, 72].

Despite these astounding advantages of this simple molecule, NO is surely not a universal messenger between the prokaryotic and the eukaryotic world. Though a variety of facultative anaerobe bacteria is able to utilize denitrification as an energy production pathway, the nitrogen-containing molecules only serve as alternative electron acceptors in the absence of oxygen. Also in the case of *N. gonorrhoeae*, the best-studied example of this microscale NO-dependent communication, nitrite reduction to NO is only seen under oxygen-limited conditions, when the copper-containing enzyme AniA is expressed as one of the main proteins induced by anaerobic conditions [73, 74]. Such anaerobic growth conditions mimic the natural habitat of gonococci, where wide areas of the genital tract are low in oxygen.

Interestingly, the relevance of this NO-mediated microscale communication for successful colonization of the genital tract is underscored by an ongoing natural experiment: *N. meningitidis*, the closest relative of gonococci, normally colonizes the highly oxygenated environment of the human nasopharynx (rather than the genital tract as for gonococci). Strikingly, recent years have seen the emergence of a *N. meningitidis* strain that is transmitted by sexual contact and that efficiently colonizes the human genital tract [75, 76]. As a major adaptation to its new lifestyle, this pathotype of the meningococcus has acquired a 3.8 kb gonococcal genome fragment encoding the nitrite reductase AniA, strongly suggesting that AniA-produced NO turns these meningococci now also into efficient colonizers of the genital tract.

## Conclusion

The last decades have seen a tremendous growth in our molecular understanding of adhesin-mediated microbe-host cell interactions and their contribution to bacterial pathogenesis. Unfortunately, these insights have not yet translated into tangible results in the fight against pathogenic bacteria. Clearly, strategies to combat pathogens by blocking their adhesive functions, and thereby compromising their ability to communicate with the host, are feasible and can reduce the prevalence of specific pathogens [77–79] (Table 1). However, such anti-adhesive approaches using soluble ligand mimetics or blocking antibodies are by design highly focused on a single virulence factor of one particular pathogenic bacterium [80]. This high precision comes at a price, as the targeted adhesin could be variably expressed, modified, or replaced by an alternative adhesin, rendering a highly selective anti-adhesive approach vulnerable to pathogen evasion mechanisms. Besides these scientific challenges, the rather limited scope of potential anti-adhesive therapies further reduces economic incentives for commercial enterprises, which are already reluctant to invest even in broad-spectrum antibiotics. Also, in the face of the global spread of multidrug-resistant bacterial strains, the finding that the unpretentious and widely occurring metabolite NO is involved in cross-kingdom communication between pathogens and human tissues opens up interesting novel treatment possibilities. Indeed, NO triggers a well-characterized signaling cascade in human cells, which can be addressed by a panel of established pharmacological inhibitors. For example, topical application of sGC antagonists at the site of infection could counteract the bacterial suppression of epithelial exfoliation and thus not only hamper the infection process, but also interrupt bacterial transmission chains. This might be a viable option in sexually transmitted diseases, such as gonorrhea, where potential transmission events do not go unnoticed and immediate post-exposure treatment appears feasible. It is intriguing to envision a future, where certain infectious diseases might be prevented by manipulating NO-triggered signaling processes in the human host. Such a host-centered treatment could never be subverted by bacterial resistance development, and by severing the delicate NO-based communication line between specialized pathogens and their target tissue, it could stop the infection process in its tracks at the initial colonization of the human mucosa.

## References

[1] Hauck CR, Meyer TF (2003). “Small“talk: Opa proteins as mediators of Neisseria-host cell communication. Curr Opin Microbiol.

[2] Tchoupa AK, Lichtenegger S, Reidl J, Hauck CR (2015). Outer membrane protein P1 is the CEACAM-binding adhesin of *Haemophilus influenzae*. Mol Microbiol.

[3] Jacobsen T, Bardiaux B, Francetic O, Izadi-Pruneyre N, Nilges M (2020). Structure and function of minor pilins of type IV pili. Med Microbiol Immunol.

[4] Piepenbrink KH, Sundberg EJ (2016). Motility and adhesion through type IV pili in Gram-positive bacteria. Biochem Soc Trans.

[5] Mu XQ, Bullitt E (2006). Structure and assembly of P-pili: a protruding hinge region used for assembly of a bacterial adhesion filament. Proc Natl Acad Sci USA.

[6] Roberts JA, Marklund BI, Ilver D, Haslam D, Kaack MB, Baskin G (1994). The Gal(alpha 1-4)Gal-specific tip adhesin of *Escherichia coli* P-fimbriae is needed for pyelonephritis to occur in the normal urinary tract. Proc Natl Acad Sci USA.

[7] Wurpel DJ, Beatson SA, Totsika M, Petty NK, Schembri MA (2013). Chaperone-usher fimbriae of *Escherichia coli*. PLoS ONE.

[8] Day CJ, Lo AW, Hartley-Tassell LE, Argente MP, Poole J, King NP, (2021). Discovery of bacterial fimbria-glycan interactions using whole-cell recombinant *Escherichia coli* Expression. mBio.

[9] Kau AL, Hunstad DA, Hultgren SJ (2005). Interaction of uropathogenic *Escherichia coli* with host uroepithelium. Curr Opin Microbiol.

[10] Wu XR, Sun TT, Medina JJ (1996). In vitro binding of type 1-fimbriated *Escherichia coli* to uroplakins Ia and Ib: relation to urinary tract infections. Proc Natl Acad Sci USA.

[11] Lee G (2011). Uroplakins in the lower urinary tract. Int Neurourol J.

[12] Sauer MM, Jakob RP, Eras J, Baday S, Eris D, Navarra G (2016). Catch-bond mechanism of the bacterial adhesin FimH. Nat Commun.

[13] Sokurenko EV, Vogel V, Thomas WE (2008). Catch-bond mechanism of force-enhanced adhesion: counterintuitive, elusive, but widespread?. Cell Host Microbe.

[14] Wang H, Min G, Glockshuber R, Sun TT, Kong XP (2009). Uropathogenic *E. coli* adhesin-induced host cell receptor conformational changes: implications in transmembrane signaling transduction. J Mol Biol.

[15] Thumbikat P, Berry RE, Zhou G, Billips BK, Yaggie RE, Zaichuk T (2009). Bacteria-induced uroplakin signaling mediates bladder response to infection. PLoS Pathog.

[16] Martinez JJ, Mulvey MA, Schilling JD, Pinkner JS, Hultgren SJ (2000). Type 1 pilus-mediated bacterial invasion of bladder epithelial cells. EMBO J.

[17] Martinez JJ, Hultgren SJ (2002). Requirement of Rho-family GTPases in the invasion of Type 1-piliated uropathogenic *Escherichia coli*. Cell Microbiol.

[18] Khare B, V L Narayana S (2017). Pilus biogenesis of Gram-positive bacteria: roles of sortases and implications for assembly. Protein Sci.

[19] Schneewind O, Missiakas D (2014). Sec-secretion and sortase-mediated anchoring of proteins in Gram-positive bacteria. Biochim Biophys Acta.

[20] Dramsi S, Caliot E, Bonne I, Guadagnini S, Prevost MC, Kojadinovic M (2006). Assembly and role of pili in group B streptococci. Mol Microbiol.

[21] Kreikemeyer B, Nakata M, Oehmcke S, Gschwendtner C, Normann J, Podbielski A (2005). *Streptococcus pyogenes* collagen type I-binding Cpa surface protein. expression profile, binding characteristics, biological functions, and potential clinical impact. J Biol Chem.

[22] Nelson AL, Ries J, Bagnoli F, Dahlberg S, Falker S, Rounioja S (2007). RrgA is a pilus-associated adhesin in *Streptococcus pneumoniae*. Mol Microbiol.

[23] Izore T, Contreras-Martel C, El Mortaji L, Manzano C, Terrasse R, Vernet T (2010). Structural basis of host cell recognition by the pilus adhesin from *Streptococcus pneumoniae*. Structure.

[24] Hoiczyk E, Roggenkamp A, Reichenbecher M, Lupas A, Heesemann J (2000). Structure and sequence analysis of *Yersinia* YadA and *Moraxella* UspAs reveal a novel class of adhesins. EMBO J.

[25] Linke D, Riess T, Autenrieth IB, Lupas A, Kempf VA (2006). Trimeric autotransporter adhesins: variable structure, common function. Trends Microbiol.

[26] El Tahir Y, Skurnik M (2001). YadA, the multifaceted *Yersinia* adhesin. Intl. J Med Microbiol.

[27] Comanducci M, Bambini S, Brunelli B, Adu-Bobie J, Arico B, Capecchi B (2002). NadA, a novel vaccine candidate of *Neisseria meningitidis*. J Exp Med.

[28] Conners R, Hill DJ, Borodina E, Agnew C, Daniell SJ, Burton NM (2008). The *Moraxella* adhesin UspA1 binds to its human CEACAM1 receptor by a deformable trimeric coiled-coil. EMBO J.

[29] Riess T, Andersson SG, Lupas A, Schaller M, Schafer A, Kyme P (2004). *Bartonella* adhesin a mediates a proangiogenic host cell response. J Exp Med.

[30] Bentancor LV, Camacho-Peiro A, Bozkurt-Guzel C, Pier GB, Maira-Litran T (2012). Identification of Ata, a multifunctional trimeric autotransporter of *Acinetobacter baumannii*. J Bacteriol.

[31] Schulze-Koops H, Burkhardt H, Heesemann J, von der Mark K, Emmrich F (1992). Plasmid-encoded outer membrane protein YadA mediates specific binding of enteropathogenic *Yersiniae* to various types of collagen. Infect Immun.

[32] Tertti R, Skurnik M, Vartio T, Kuusela P (1992). Adhesion protein YadA of *Yersinia* species mediates binding of bacteria to fibronectin. Infect Immun.

[33] Eitel J, Heise T, Thiesen U, Dersch P (2005). Cell invasion and IL-8 production pathways initiated by YadA of *Yersinia pseudotuberculosis* require common signalling molecules (FAK, c-Src, Ras) and distinct cell factors. Cell Microbiol.

[34] Heise T, Dersch P (2006). Identification of a domain in *Yersinia* virulence factor YadA that is crucial for extracellular matrix-specific cell adhesion and uptake. Proc Natl Acad Sci USA.

[35] Owen KA, Thomas KS, Bouton AH (2007). The differential expression of *Yersinia pseudotuberculosis* adhesins determines the requirement for FAK and/or Pyk2 during bacterial phagocytosis by macrophages. Cell Microbiol.

[36] Hamburger ZA, Brown MS, Isberg RR, Bjorkman PJ (1999). Crystal structure of invasin: a bacterial integrin-binding protein. Science.

[37] Alrutz MA, Isberg RR (1998). Involvement of focal adhesion kinase in invasin-mediated uptake. Proc Natl Acad Sci USA.

[38] Eitel J, Dersch P (2002). The YadA protein of *Yersinia pseudotuberculosis* mediates high-efficiency uptake into human cells under environmental conditions in which invasin is repressed. Infect Immun.

[39] Keller B, Muhlenkamp M, Deuschle E, Siegfried A, Mossner S, Schade J (2015). *Yersinia enterocolitica* exploits different pathways to accomplish adhesion and toxin injection into host cells. Cell Microbiol.

[40] Lecuit M, Dramsi S, Gottardi C, Fedor-Chaiken M, Gumbiner B, Cossart P (1999). A single amino acid in E-cadherin responsible for host specificity towards the human pathogen *Listeria monocytogenes*. EMBO J.

[41] Mengaud J, Ohayon H, Gounon P, Mege RM, Cossart P (1996). E-cadherin is the receptor for internalin, a surface protein required for entry of *L. monocytogenes* into epithelial cells. Cell.

[42] Schubert W-D, Urbanke C, Ziehm T, Beier V, Machner MP, Domann E (2002). Structure of internalin, a major invasion protein of *Listeria monocytogenes*, in complex with its human receptor E-cadherin. Cell.

[43] Bonazzi M, Veiga E, Pizarro-Cerda J, Cossart P (2008). Successive post-translational modifications of E-cadherin are required for InlA-mediated internalization of *Listeria monocytogenes*. Cell Microbiol.

[44] Veiga E, Cossart P (2005). *Listeria* hijacks the clathrin-dependent endocytic machinery to invade mammalian cells. Nat Cell Biol.

[45] Anderton JM, Rajam G, Romero-Steiner S, Summer S, Kowalczyk AP, Carlone GM (2007). E-cadherin is a receptor for the common protein pneumococcal surface adhesin A (PsaA) of *Streptococcus pneumoniae*. Microb Pathogenesis.

[46] Eshghi A, Gaultney RA, England P, Brule S, Miras I, Sato H (2019). An extracellular *Leptospira interrogans* leucine-rich repeat protein binds human E- and VE-cadherins. Cell Microbiol.

[47] Evangelista K, Franco R, Schwab A, Coburn J (2014). *Leptospira interrogans* binds to cadherins. PLoS Negl Trop Dis.

[48] Fardini Y, Wang X, Temoin S, Nithianantham S, Lee D, Shoham M (2011). *Fusobacterium nucleatum* adhesin FadA binds vascular endothelial cadherin and alters endothelial integrity. Mol Microbiol.

[49] Rubinstein MR, Wang X, Liu W, Hao Y, Cai G, Han YW (2013). *Fusobacterium nucleatum* promotes colorectal carcinogenesis by modulating E-cadherin/beta-catenin signaling via its FadA adhesin. Cell Host Microbe.

[50] Castellarin M, Warren RL, Freeman JD, Dreolini L, Krzywinski M, Strauss J (2012). *Fusobacterium nucleatum* infection is prevalent in human colorectal carcinoma. Genome Res.

[51] Kostic AD, Chun E, Robertson L, Glickman JN, Gallini CA, Michaud M (2013). *Fusobacterium nucleatum* potentiates intestinal tumorigenesis and modulates the tumor-immune microenvironment. Cell Host Microbe.

[52] Kostic AD, Gevers D, Pedamallu CS, Michaud M, Duke F, Earl AM (2012). Genomic analysis identifies association of *Fusobacterium* with colorectal carcinoma. Genome Res.

[53] Hill DJ, Virji M (2003). A novel cell-binding mechanism of *Moraxella catarrhalis* ubiquitous surface protein UspA: specific targeting of the N-domain of carcinoembryonic antigen-related cell adhesion molecules by UspA1. Mol Microbiol.

[54] Hill DJ, Whittles C, Virji M (2012). A novel group of *Moraxella catarrhalis* UspA proteins mediates cellular adhesion via CEACAMs and vitronectin. PLoS ONE.

[55] Koniger V, Holsten L, Harrison U, Busch B, Loell E, Zhao Q (2016). *Helicobacter pylori* exploits human CEACAMs via HopQ for adherence and translocation of CagA. Nat Microbiol.

[56] Berger CN, Billker O, Meyer TF, Servin AL, Kansau I (2004). Differential recognition of members of the carcinoembryonic antigen family by Afa/Dr adhesins of diffusely adhering *Escherichia coli* (Afa/Dr DAEC). Mol Microbiol.

[57] van Sorge NM, Bonsor DA, Deng L, Lindahl E, Schmitt V, Lyndin M (2021). Bacterial protein domains with a novel Ig-like fold target human CEACAM receptors. EMBO J.

[58] Tchoupa AK, Schuhmacher T, Hauck CR (2014). Signaling by epithelial members of the CEACAM family - mucosal docking sites for pathogenic bacteria. Cell Commun Signal.

[59] Muenzner P, Rohde M, Kneitz S, Hauck CR (2005). CEACAM engagement by human pathogens enhances cell adhesion and counteracts bacteria-induced detachment of epithelial cells. J Cell Biol.

[60] Muenzner P, Bachmann V, Hentschel J, Zimmermann W, Hauck CR (2010). Human-restricted bacterial pathogens block shedding of epithelial cells by stimulating integrin activation. Science.

[61] Muenzner P, Kengmo Tchoupa A, Klauser B, Brunner T, Putze J, Dobrindt U (2016). Uropathogenic *E. coli* exploit CEA to promote colonization of the urogenital tract mucosa. PLoS Pathog.

[62] Hauck CR, Borisova M, Muenzner P (2012). Exploitation of integrin function by pathoghenic microbes. Curr Opin Cell Biol.

[63] Muenzner P, Hauck CR (2020). Neisseria gonorrhoeae blocks epithelial exfoliation by nitric-oxide-mediated metabolic cross talk to promote colonization in mice. Cell Host Microbe.

[64] Ignarro LJ (1990). Biosynthesis and metabolism of endothelium-derived nitric oxide. Ann Rev Pharmacol Toxicol.

[65] Cinelli MA, Do HT, Miley GP, Silverman RB (2020). Inducible nitric oxide synthase: Regulation, structure, and inhibition. Med Res Rev.

[66] Grisham MB, Jourd’Heuil D, Wink DA (1999). Nitric oxide. I. Physiological chemistry of nitric oxide and its metabolites:implications in inflammation. Am J Physiol.

[67] Furchgott RF, Jothianandan D (1991). Endothelium-dependent and -independent vasodilation involving cyclic GMP: relaxation induced by nitric oxide, carbon monoxide and light. Blood Vessels.

[68] Koh A, De Vadder F, Kovatcheva-Datchary P, Backhed F (2016). From dietary fiber to host physiology: short-chain fatty acids as key bacterial metabolites. Cell.

[69] Schroeder BO, Backhed F (2016). Signals from the gut microbiota to distant organs in physiology and disease. Nat Med.

[70] Luu M, Weigand K, Wedi F, Breidenbend C, Leister H, Pautz S (2018). Regulation of the effector function of CD8(+) T cells by gut microbiota-derived metabolite butyrate. Sci Rep.

[71] Smith PM, Howitt MR, Panikov N, Michaud M, Gallini CA, Bohlooly YM (2013). The microbial metabolites, short-chain fatty acids, regulate colonic Treg cell homeostasis. Science.

[72] Alderton WK, Cooper CE, Knowles RG (2001). Nitric oxide synthases: structure, function and inhibition. Biochem J.

[73] Boulanger MJ, Murphy ME (2002). Crystal structure of the soluble domain of the major anaerobically induced outer membrane protein (AniA) from pathogenic *Neisseria*: a new class of copper-containing nitrite reductases. J Mol Biol.

[74] Mellies J, Jose J, Meyer TF (1997). The *Neisseria gonorrhoeae* gene aniA encodes an inducible nitrite reductase. Mol Gen Genet.

[75] Bazan JA, Peterson AS, Kirkcaldy RD, Briere EC, Maierhofer C, Turner AN (2016). Notes from the field: increase in *Neisseria meningitidis*-associated urethritis among men at two sentinel clinics - Columbus, Ohio, and Oakland County, Michigan, 2015. Morbidity Mortal Wkly Rep.

[76] Tzeng YL, Bazan JA, Turner AN, Wang X, Retchless AC, Read TD (2017). Emergence of a new *Neisseria meningitidis* clonal complex 11 lineage 11.2 clade as an effective urogenital pathogen. Proc Natl Acad Sci USA.

[77] Alvarez Dorta D, Sivignon A, Chalopin T, Dumych TI, Roos G, Bilyy RO (2016). The antiadhesive strategy in Crohn’s disease: orally active mannosides to decolonize pathogenic *Escherichia coli* from the gut. Chembiochem.

[78] Leonard AC, Petrie LE, Cox G (2019). Bacterial anti-adhesives: inhibition of *Staphylococcus aureus* nasal colonization. ACS Infect Dis.

[79] Spaulding CN, Klein RD, Ruer S, Kau AL, Schreiber HL, Cusumano ZT (2017). Selective depletion of uropathogenic *E. coli* from the gut by a FimH antagonist. Nature.

[80] Spaulding CN, Klein RD, Schreiber HL, Janetka JW, Hultgren SJ (2018). Precision antimicrobial therapeutics: the path of least resistance?. NPJ Biofilms Microbiomes.

[81] Mandlik A, Swierczynski A, Das A, Ton-That H (2007). *Corynebacterium diphtheriae* employs specific minor pilins to target human pharyngeal epithelial cells. Mol Microbiol.

[82] Ohlsson J, Jass J, Uhlin BE, Kihlberg J, Nilsson UJ (2002). Discovery of potent inhibitors of PapG adhesins from uropathogenic *Escherichia coli* through synthesis and evaluation of galabiose derivatives. Chembiochem.

[83] Steenhuis M, Abdallah AM, de Munnik SM, Kuhne S, Sterk GJ, van den Berg van Saparoea B (2019). Inhibition of autotransporter biogenesis by small molecules. Mol Microbiol.

[84] Brooks MJ, Sedillo JL, Wagner N, Wang W, Attia AS, Wong H (2008). *Moraxella catarrhalis* binding to host cellular receptors is mediated by sequence-specific determinants not conserved among all UspA1 protein variants. Infect Immun.

[85] Chen T, Grunert F, Medina-Marino A, Gotschlich EC (1997). Several carcinoembryonic antigens (CD66) serve as receptors for gonococcal opacity proteins. J Exp Med.

[86] Virji M, Watt SM, Barker S, Makepeace K, Doyonnas R (1996). The N-domain of the human CD66a adhesion molecule is a target for Opa proteins of *Neisseria meningitidis* and *Neisseria gonorrhoeae*. Mol Microbiol.

[87] Scietti L, Sampieri K, Pinzuti I, Bartolini E, Benucci B, Liguori A (2016). Exploring host-pathogen interactions through genome wide protein microarray analysis. Sci Rep.

[88] Bajaj M, Mamidyala SK, Zuegg J, Begg SL, Ween MP, Luo Z (2015). Discovery of novel pneumococcal surface antigen A (PsaA) inhibitors using a fragment-based drug design approach. ACS Chem Biol.

[89] Obaidullah AJ, Ahmed MH, Kitten T, Kellogg GE (2018). Inhibiting pneumococcal surface antigen A (PsaA) with small molecules discovered through virtual screening: steps toward validating a potential target for *Streptococcus pneumoniae*. Chem Biodiv.

[90] Dersch P, Isberg RR (1999). A region of the Yersinia pseudotuberculosis invasin protein enhances integrin-mediated uptake into mammalian cells and promotes self-association. EMBO J.

[91] Isberg RR, Leong JM (1990). Multiple beta 1 chain integrins are receptors for invasin, a protein that promotes bacterial penetration into mammalian cells. Cell.

